# Characterizing Brain Structures and Remodeling after TBI Based on Information Content, Diffusion Entropy

**DOI:** 10.1371/journal.pone.0076343

**Published:** 2013-10-15

**Authors:** Niloufar Fozouni, Michael Chopp, Siamak P. Nejad-Davarani, Zheng Gang Zhang, Norman L. Lehman, Steven Gu, Yuji Ueno, Mei Lu, Guangliang Ding, Lian Li, Jiani Hu, Hassan Bagher-Ebadian, David Hearshen, Quan Jiang

**Affiliations:** 1 Department of Neurology, Henry Ford Hospital, Detroit, Michigan, United States of America; 2 Department of Biostatistics and Research Epidemiology, Henry Ford Hospital, Detroit, Michigan, United States of America; 3 Department of Pathology, Henry Ford Hospital, Detroit, Michigan, United States of America; 4 Department of Radiology, Henry Ford Hospital, Detroit, Michigan, United States of America; 5 MR Center, Harper Hospita, Detroit, Michigan, United States of America; 6 Department of Physics, Oakland University, Rochester, Michigan, United States of America; University G. D'Annunzio, Italy

## Abstract

**Background:**

To overcome the limitations of conventional diffusion tensor magnetic resonance imaging resulting from the assumption of a Gaussian diffusion model for characterizing voxels containing multiple axonal orientations, Shannon's entropy was employed to evaluate white matter structure in human brain and in brain remodeling after traumatic brain injury (TBI) in a rat.

**Methods:**

Thirteen healthy subjects were investigated using a Q-ball based DTI data sampling scheme. FA and entropy values were measured in white matter bundles, white matter fiber crossing areas, different gray matter (GM) regions and cerebrospinal fluid (CSF). Axonal densities' from the same regions of interest (ROIs) were evaluated in Bielschowsky and Luxol fast blue stained autopsy (n = 30) brain sections by light microscopy. As a case demonstration, a Wistar rat subjected to TBI and treated with bone marrow stromal cells (MSC) 1 week after TBI was employed to illustrate the superior ability of entropy over FA in detecting reorganized crossing axonal bundles as confirmed by histological analysis with Bielschowsky and Luxol fast blue staining.

**Results:**

Unlike FA, entropy was less affected by axonal orientation and more affected by axonal density. A significant agreement (r = 0.91) was detected between entropy values from in vivo human brain and histologically measured axonal density from post mortum from the same brain structures. The MSC treated TBI rat demonstrated that the entropy approach is superior to FA in detecting axonal remodeling after injury. Compared with FA, entropy detected new axonal remodeling regions with crossing axons, confirmed with immunohistological staining.

**Conclusions:**

Entropy measurement is more effective in distinguishing axonal remodeling after injury, when compared with FA. Entropy is also more sensitive to axonal density than axonal orientation, and thus may provide a more accurate reflection of axonal changes that occur in neurological injury and disease.

## Introduction

Diffusion Tensor Imaging (DTI), developed more than a decade ago [1], has been successfully used for the study of brain anatomy and in clinical neurodiagnostics, the latter especially for disease processes involving the white matter, such as multiple sclerosis (MS) [2], [3], amyotrophic lateral sclerosis (ALS) [4], cerebral ischemia [5], [6], brain tumors [7], [8], and head trauma [9], [10], [11].

The diffusivity from traditional DTI is derived from a symmetric rank-2, positive tensor[12]. The most important indices that can be derived from DTI are diffusivity, Relative Anisotropy (RA), Fractional Anisotropy (FA), color-coded fiber direction maps and 3-D fiber tractography [13]. Amongst these, FA is the most widely used index for quantitatively characterizing neurodegenerative conditions, such as aging, Parkinson's disease, developmental disorders [14], and white matter disease [15], [16], [17].

Despite its popular application, conventional DTI has shortcomings resulting from its two underlying assumptions. First, the use of a ‘single’ diffusion tensor for characterizing a pixel volume, which may contain thousands of tissue components, results in a diffusion tensor representing only an average of these multiple tissue components (compartments). Examples of DTI model failure in analyzing areas of fiber crossing in white matter have been documented [18], [19]. For example, areas of white matter containing two (or more) fiber systems passing within the same pixel appear hypo-intense in FA. Conventional DTI thus inappropriately yields low FA in these crossing fiber regions. Also, FA is insensitive in detecting axonal density in gray matter due to the relatively random fiber orientation distribution and lower axonal density. The second shortcoming of the DTI model results from its assumption that water diffusion in white matter follows a Gaussian distribution [20]. It has been shown that diffusion heterogeneity of compartments may result in non-Gaussian diffusion [21], [22], [23], [24], [25]. Indeed, recent experimental results have demonstrated non-Gaussian diffusion in white matter, especially with high b-values, [21], [23], [26], [27]. Collectively, the foregoing observations indicate the unreliability of the conventional DTI model in addressing the non-Gaussian diffusion in the brain.

To overcome these limitations of DTI, Q-space diffusion tensor imaging (Q-DTI), such as high angular resolution diffusion imaging (HARDI)[18], was developed to resolve intravoxel fiber crossing [18], [28], [29], [30], [31], [32], [33]. In contrast to conventional DTI, free model Q-DTI can measure diffusion function directly, based on the Fourier relationship between diffusion signal and diffusion function within each voxel, without relying on a superimposed model. This model-independent approach can resolve axonal fiber crossing in the brain [29], [34]. Two types of information are obtained by Q-DTI: directionality and distance – the mean free path (from the second moment of the probability distribution function (PDF) of path lengths), and higher moments. The various approaches to the assessment of directionality, such as diffusion spectrum imaging (DSI) [34], q-ball [29], persistent angular structure MRI (PASMRI) [19], have been previously described. These methods are more focused on the directionality of fiber crossing, however little attention is paid to the mean free path and quantification[33], [35], [36]. In this study, we investigated the diffusion entropy approach for quantitatively characterizing uncertainty of mean free path from diffusion-weighted magnetic resonance imaging. This approach relies on the application of Shannon's entropy and calculates entropy based on the probability distribution of random variables [37]. We demonstrate that entropy measurements can better characterize axonal properties in locations containing multiple diffusion orientations, resulting in improved image contrast when compared with FA maps. We also test the ability of diffusion entropy in white matter reorganization with crossing axonal bundles after TBI.

### Theory

Shannon's entropy is a measure of uncertainty or randomness based on information theory [37]. Entropy yields the information content of the data: more information reflects a greater reduction of uncertainty, or higher certainty. In this study, we hypothesized that entropy changes according to brain tissue types. Less structure in isotropic tissues such as CSF would indicate the presence of more equal states and therefore higher certainty or lower entropy, while in tissues with higher compartment density and complexity there exists more information content yielding higher uncertainty or higher entropy.

Shannon's entropy (*H*) is evaluated via the equation:
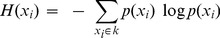
(1)where *p(x_i_)* is the probability that *x_i_* is in the state *x_i_*, here we used *x_i_* to represent the attenuation value, *k* is all possible diffusion gradient directions, and *p(x_i_)* shows probability of repetition of specific attenuation in special gradient direction. The logarithm is based 2 and entropy can be articulated in bits. *p(x_i_)* log *p(x_i_)* becomes 0 when *p* = 0. Since CSF is essentially isotropic in diffusion, diffusion in CSF is independent of gradient directions, yielding equal probabilities in all directions, resulting in diminished values of entropy *H*. This is not however the case for gray or white matter [36].

## Materials and Methods

Thirteen volunteers aged 18–55 years (9 males and 4 females; 34.9±10 years, median 32 years) with no history of neurological or psychological disorders participated in this study. Ten subjects were enrolled for the comparison between entropy and FA and the remaining three for the investigation of the effects of numbers of gradient directions on entropy. In our case study, a rat with MSC treatment after TBI was also studied [10]. The crossing axon bundles in the remodeling areas of the TBI animal brain had been identified [10] and to be used to test the ability of diffusion entropy. All studies were conducted within the guidelines of the internal review board and IACUC of our institution.

### MRI Measurements in Human Subjects

MR images were obtained with a GE 3T MRI scanner utilizing an eight-channel head coil. The subject's head was comfortably secured in the head coil using foam padding. Disposable earplugs were provided to minimize the subject's exposure to instrument noise during scanning. T2 and DTI MRI measurements were obtained using the following parameters.

#### T_2_ measurements

T_2_ was obtained using a fast spin-echo sequence. A TR value of 2.5 seconds was utilized with effective echo times of 14 and 120 milliseconds. Images were produced with a 24 cm field of view, 4 mm slice thickness, 32 slices, and a 512×512 matrix.

#### DTI measurements

DTI was acquired using a pulsed gradient spin-echo echo-planar sequence with a TE/TR ratio of 92/10 ms, a 24 cm field of view, 96×96 imaging matrix, 2.6 mm slice thickness, b-value of 1500 s/mm^2^, 15–90 directions uniformly distributed in space depending on the experiments, and 1 average and 6 b = 0 T_2_ weighted images.

### Animal Model and Experiment

Traumatic brain injury (TBI) *via* controlled cortical impact [38], [39] was induced in a male Wistar rat (n = 1). Seven days after TBI a collagen scaffold suffused with 3×10^6^ human bone marrow stromal cells (hMSCs) was implanted into the core of the lesion [10]. During surgery and during transplant of hMSCs, animals were anesthetized with 3.5% halothane and then maintained with 1.0∼2.0% halothane in N_2_O:O_2_ (2∶1).

### MRI Measurements in 7 Tesla Animal System

MRI measurements were performed using a Varian 7 Tesla MRI scanner (Palo Alto, CA). A 12 cm bore actively shielded gradient coil set, capable of producing magnetic field gradients up to 20 gauss/cm was used. A saddle radio-frequency (RF) coil was used as the transmitter and a surface coil as the receiver. The animal was sacrificed at 6 weeks after TBI and *ex vivo* DTI was performed one day after death.

#### *Ex vivo* Q-space DTI measurement

Q-ball based DTI was performed using a pulsed gradient spin-echo sequence. The FOV was 32 mm; four average, 128×128 imaging matrix, 1 mm slice thickness with 16 slices, TR = 1.5 s, TE = 38 ms, δ = 12 ms, Δ = 20 ms, 128 diffusion attenuated directions with b = 1500 s/mm^2^ in each slice [19], for a total acquisition time of about 27 hours.

### Histological Staining

Human brain autopsy tissue samples (n = 30) and animal brain (n = 1) were studied for axonal analysis. To identify cerebral white matter properties, immunohistochemistry was performed on formalin-fixed, paraffin-embedded coronal brain sections (6 µm). Axonal density and orientation were examined using a combined Nissl/silver-staining method (Bielschowsky staining) [40]. Double Bielschowsky and Luxol fast blue [41], staining was used to demonstrate axons and myelin, respectively. For Bielschowsky staining, slides were placed in 20% silver nitrate in the dark, then ammonium hydroxide was added to stain slides until the tissues turned brown with a gold background, and were then treated with sodium thiosulfate. Slides were then stained in Luxol fast blue solution, washed in 95% alcohol, and then placed in lithium carbonate. Nuclei are colorless; myelin is blue and axons appear black.

### Data Analysis

**Figure 1:**
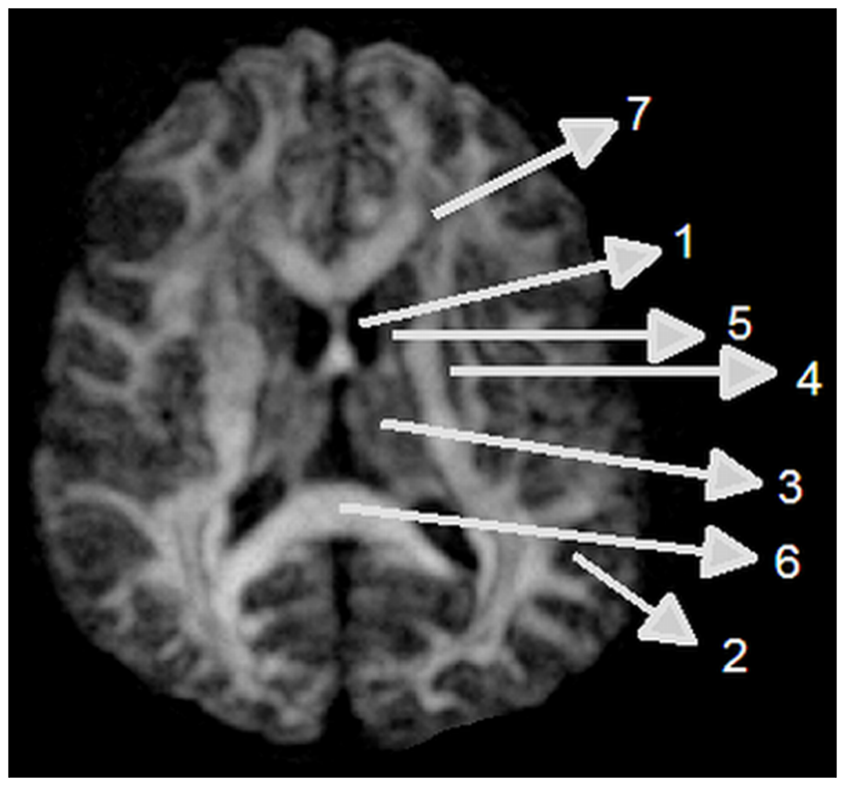
Regions of interest for entropy and FA measurements: 1, CSF; 2, cortical gray matter; 3, thalamus; 4, putamen; 5, caudate nucleus; 6, corpus callosum; and 7, frontal white matter.

Entropy was calculated on a voxel-by-voxel basis using equation (1) via in-house software written in Matlab (The Mathworks, Natick, MA). Fiber orientation maps from q-ball DTI data were derived using Camino [19], [42]. FA map was generated using DTIstudio Software [43]. Regions of interest (ROI) were chosen in CSF, frontal white matter with U-fiber crossing, parallel-oriented axonal fibers of the corpus callosum splenium, and, gray matter (thalamus, caudate, putamen, cortical gray matter) as shown in Fig. 1.

To perform comparisons between histological axonal density with corresponding MRI entropy and FA, five randomly chosen fields of view in each ROI of cortical gray matter, corpus callosum (CC), putamen, thalamus, and frontal white matter (FW) of histological sections were digitized using a 40× objective (Olympus BX40) via the MCID imaging system (Imaging Research, St. Catharines, Canada). Captured images were digitally level-adjusted using the public domain NIH Image analysis program version 1.42 (National Institutes of Health). Each black and white 8-bit image was binarized with the intensity threshold set at a pixel value of 170. The number of black pixels divided by the total number of pixels in the selected area was measured to represent axonal density. The average value from five fields of view in each ROI of histological sections was used in the correlation data analysis. The MRI entropy and FA were measured to correlate the histological axonal density from the same anatomic ROI. MRI analysis and histological analysis were performed by blind analysis of two persons.

ANCOVA was used to test the differences between entropy and FA (as outcome of interest) among the regions (as correlated dependent covariate) to test overall region (CC, FW) effect on the difference. The paired t-test was performed at the region level if there was an overall region effect. For evaluating the correlation between entropy, FA, and histological axonal density, we correlated the means of entropy, FA, and histological axonal density in each ROI, as exploratory analysis, since we could not get the histological data from patients alive and the MRI and histological data were from different samples. Spearman correlation coefficient was calculated to measure the correlation of entropy and FA with histological evaluation, respectively. For evaluating effects of number of gradient directions (as 15, 25, 55, 70 and 90 directions) on entropy, entropy was measured at four regions of interest (CC, FW, gray matter, and CSF). The doubly repeated measure analysis of variance (ANCOVA) was used to test the direction and region effects (as correlated covariates) on entropy measurements (outcome of interest). The analysis started with testing for the number of directions by region interaction, followed by the subgroup analysis with focus on the direction effect at each specific region, if the interaction was significant at 0.05 level. A significant interaction indicated that the direction effects varied among the regions. The optimal number of directions was selected if entropy values with minimum directions approached that measured in 90 directions.

## Results

**Figure 2:**
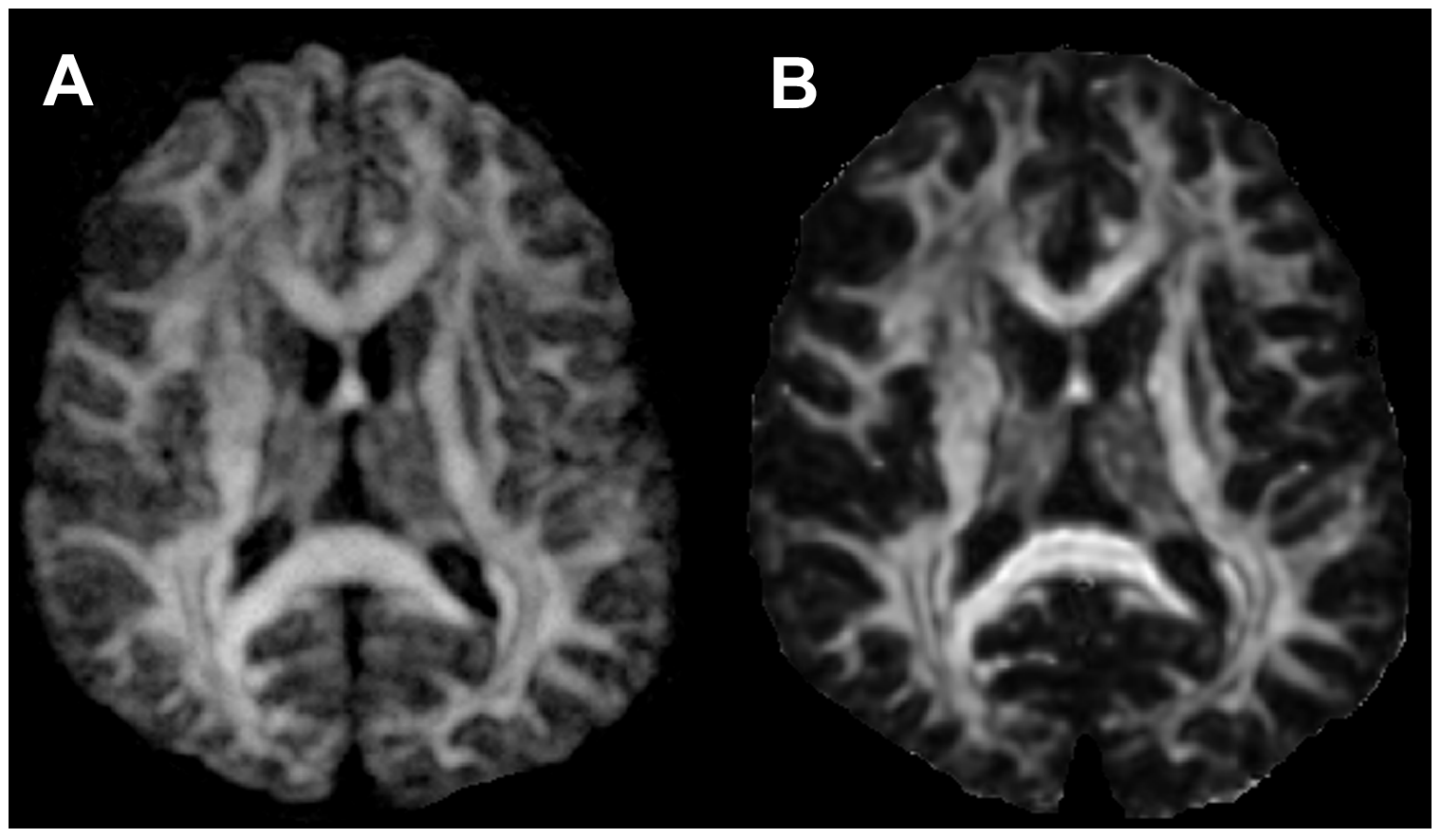
Comparison between the entropy (A) and the FA map (B) from the same subject. Gray matter is more visible in the entropy map compared to the FA map.

In the human study, the signal intensities in the entropy and FA maps showed similar patterns of intensity changes, from the highest in CC, intermediate in gray matter, and the lowest in CSF. However, entropy maps exhibited a significantly enhanced dynamic range of contrast, especially in gray matter, compared with FA maps as demonstrated in Figure 2. Also, the anatomic details of brain structure in gray matter can be much more easily identified in the entropy map compared with the more uniform unidentified dark regions seen in the FA map.

To investigate the effects of number of directions of gradients on entropy in the human study, fifteen to ninety directions of diffusion gradients were performed. The corresponding entropy values measured from the same ROIs in CC, Cortex gray matter, FW, and CSF ROIs in figure 1 were listed in the table 1. The entropy values in the CC increased with increase in number of directions from 15 to 55 and then were relatively stable from 55 to 90. Statistical results in CC exhibited significant difference (p<0.05) between 15 and 25 directions vs 90 directions, and no significant difference between 55 and 70 vs 90. In CSF ROI, entropy values decreased as number of directions increase, but significant difference was only detected between 15 vs 25.

**Table 1:** Entropy changes with number of gradients directions in different brain structures

ROIs	15 directions	25 directions	55 directions	70 directions	90 directions
	Mean (std)	Mean (std)	Mean (std)	Mean (std)	Mean (std)
WM (CC)	2.67 (0.72)	2.74 (0.55)	3.03 (0.62)	2.95 (0.7)	2.97 (0.62)
FW	1.48 (0.4)	1.45 (0.4)	1.36 (0.5)	1.45 (0.41)	1.51 (0.34)
GM (cortex)	0.85 (0.32)	0.95 (0.3)	1.13 (0.22)	1.17 (0.39)	1.35 (0.35)
CSF	1.07 (0.12)	0.99 (0.13)	0.96 (0.14)	0.93 (0.2)	0.83 (0.2)

Entropy in gray matter increased as number of directions increase from 15 to 90 and there were significant differences between 15 and 25 vs 90 and 70 as well as 15 vs 55. The entropy in FW exhibited a trend independent of the number of directions. However, a significant difference was detected only between 15 and 25 directions.

**Figure 3:**
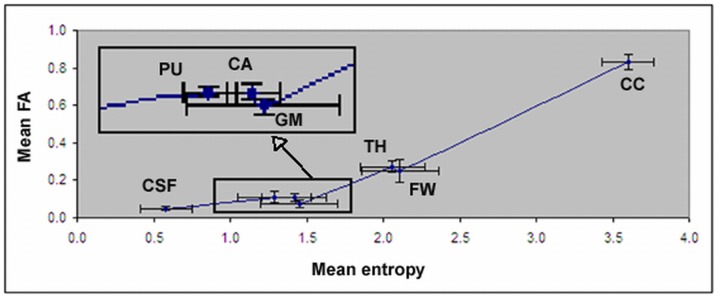
Entropy vs. FA in CSF, cerebral gray matter (GM), thalamus (TH), putamen (PU), caudate nucleus (CA), FW, and CC. The details for gray matter, putamen, caudate nucleus are enlarged from the bottom box area. Error bars indicate one standard deviation (N = 10).

Figure 3 shows a direct comparison between entropy and FA values in CSF, gray matter, and WM. CC has the largest and CSF has the smallest value in both entropy and FA maps. Using FA, it is difficult to differentiate between CSF and gray matter, and between different locations in gray matter like putamen and caudate nucleus. However, entropy exhibited a better separation between CSF and gray matter than did FA. Entropy values in different brain structures, especially in gray matter, also exhibited greater dynamic range than FA values.

To quantitatively compare the differences between entropy and FA, entropy and FA values from each ROI were measured and are listed in table 2. The distinction of mean values between CC and different locations in gray matter is more pronounced in the entropy map than in the FA map.

**Table 2:** Entropy, FA, corresponding axonal density in the ROIs displayed in Fig1.

Tissue Type in ROIs	Axonal density Mean (std)	Entropy Mean (std)	Mean% of entropy	FA, Mean (std)	Mean% of FA
WM(corpus callosum)	69.5 (4.6)	3.61 (0.17)	%100	0.83 (0.04)	%100
Frontal white matter	60.1 (1.9)	2.11 (0.25)	%59	0.25 (0.06)	%30
Thalamus	38.3 (3.0)	2.06 (0.21)	%57	0.27 (0.03)	%33
Cortex GM	24.4(1.6)	1.45 (0.25)	%40	0.08 (0.02)	%10
Caudate nucleus	N/A	1.42 (0.21)	%39	0.11 (0.02)	%13
Putamen	19.9 (4.4)	1.30 (0.24)	%36	0.11 (0.03)	%13
CSF	N/A	0.58 (0.17)	%16	0.05 (0.01)	%6

Mean% means percentage after normalization with respect to CC

To better compare the differences between entropy and FA, percentages of normalized values for entropy and FA in different regions with respect to corpus callosum were calculated as listed in the table 2. Comparing with FA, the relative values of entropy were almost doubled in FW and thalamus, tripled in putamen and caudate nucleus, and quadrupled in brain cortex, respectively.

**Figure 4:**
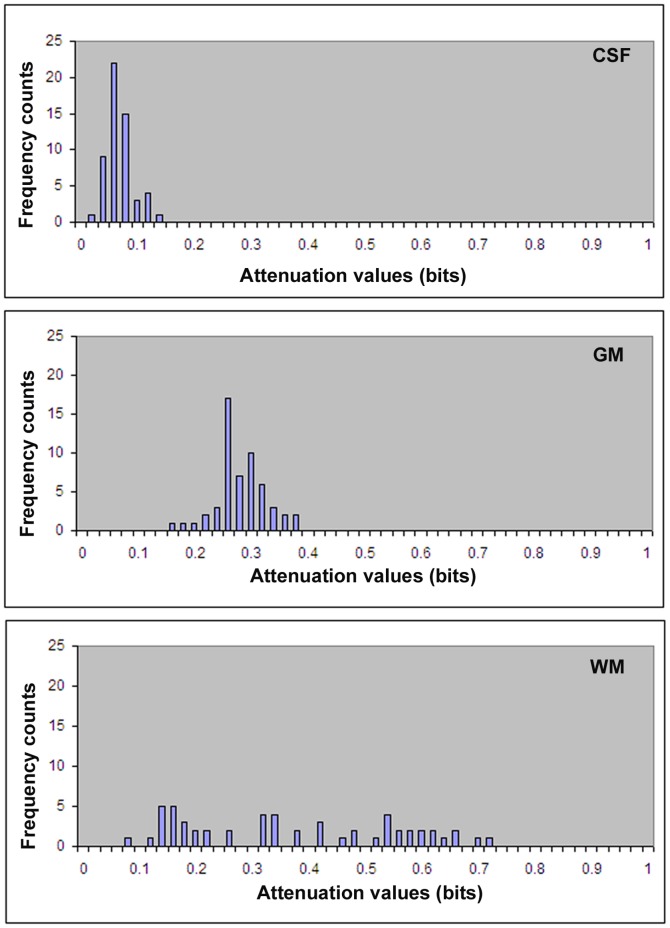
Histograms of diffusion attenuation values in CSF (top) shows high probability of occurrence of attenuation values, causing smaller values of entropy. Gray matter (middle) and white matter (bottom) show more spread of attenuation values, causing larger entropy.

Figure 4 shows histograms of diffusion attenuation values in the ROI of CSF, gray matter, and WM. The histogram of CSF shows that the probability of occurrence of attenuation values is nearly independent of the direction of gradient, which represents isotropic tissues with a high probability of occurrence in all diffusion gradients, and the entropy is close to zero. In contrast, white matter tissues show dependency of attenuation values on the direction of gradients. As noted in the histogram of white matter, the attenuation values are more evenly spread, which indicates, that as direction of the gradient changes so does information, with a higher uncertainty or higher entropy value.

**Figure 5:**
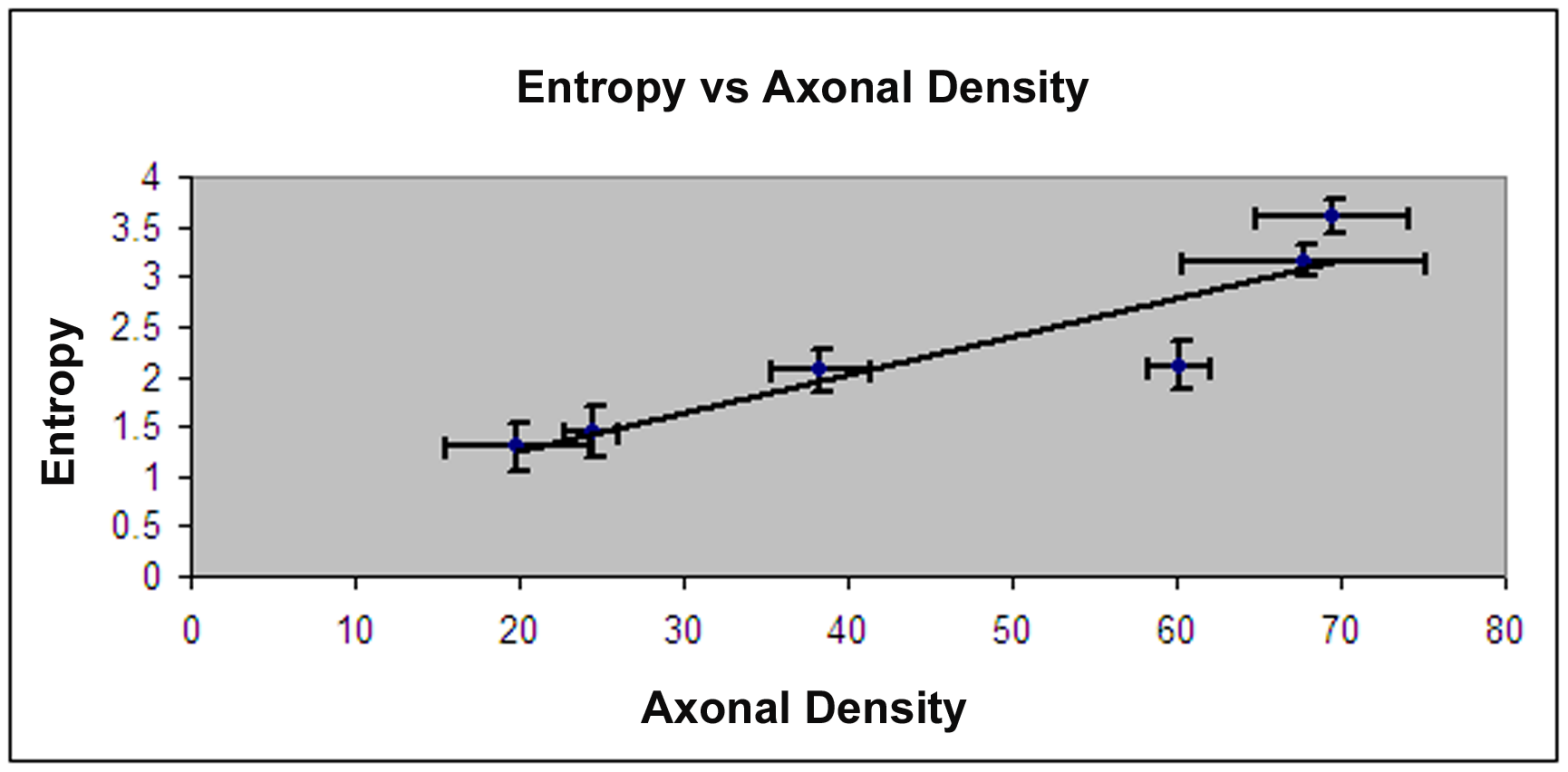
Correlation between entropy and axonal density measured from Bielschowsky and Luxol fast blue staining. Error bars indicate one standard deviation, and N = 5

In figure 5 the correlations between means of entropy values and axonal density from Bielschowsky and Luxol fast blue staining for the same ROIs are presented. The entropy values provide better correlation with the histological axonal density (r = 0.91, p = 0.04) for each region compared with FA (r = 0.86, P = 0.01).

**Figure 6:**
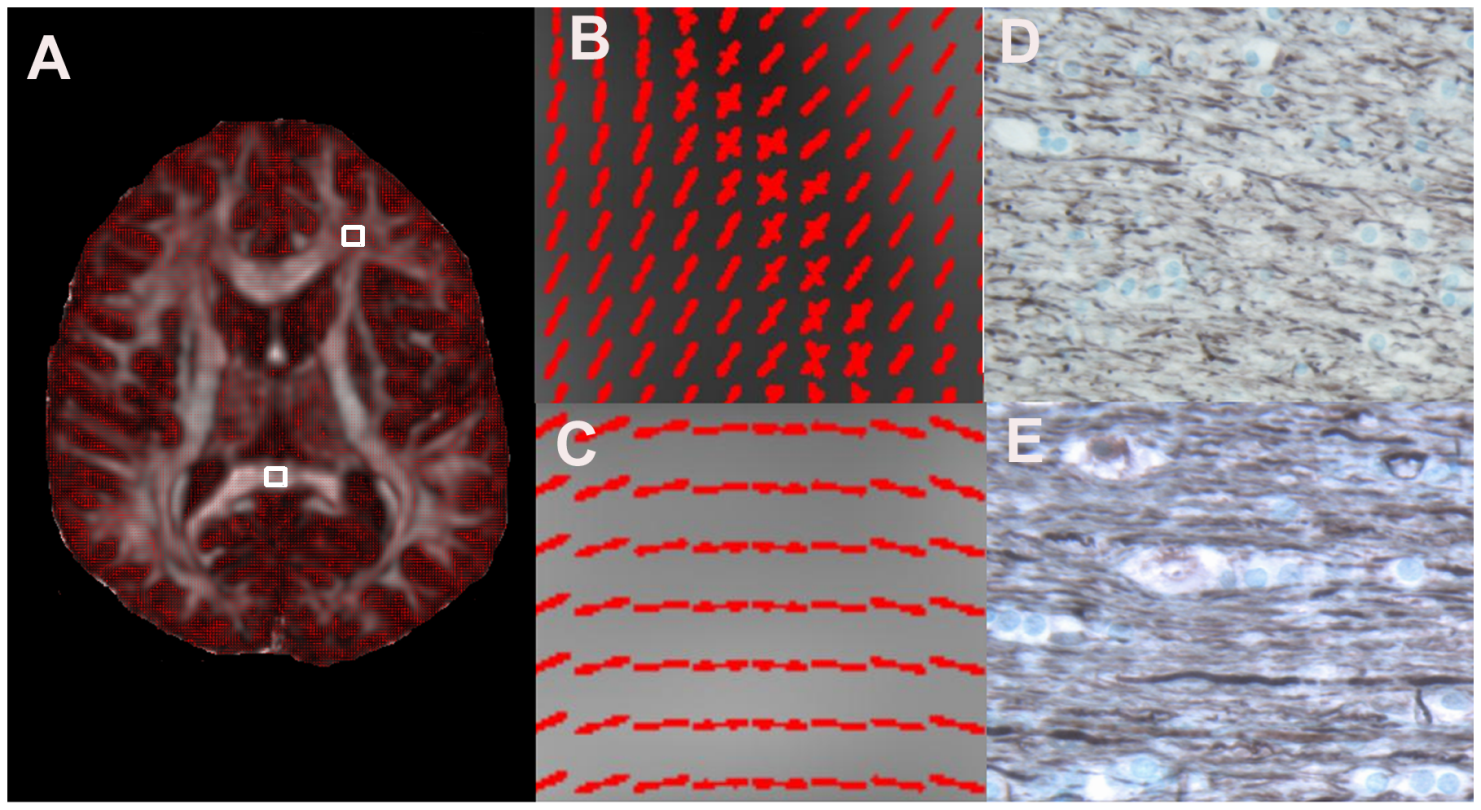
Selection of ROIs from frontal white matter (A, FW, top) and corpus callosum (A, CC, bottom), magnified of ROIs from A in FW (B) and CC (C). The corresponding Bielschowsky and Luxol fast blue staining images in FW (D), and CC (E), respectively, showing axonal density changes in these ROIs.

Figure 6 shows Q-ball fiber orientation map in CC and FW and corresponding Bielschowsky and Luxol fast blue images from human autopsy brain sections. The CC shows very dense and unidirectional axons in Bielschowsky and Luxol fast blue image (Fig 6E) consistent with the fiber orientation in Q-ball image (Fig 6C), whereas in FW axons are less dense (Fig 6 D) and show multi directionality of fibers (Fig 6B). As demonstrated in table 2, entropy reveals a decrease in magnitude in axonal density from the frontal white matter to thalamus, cortex gray matter, and putamen following the same pattern of decrease in axonal densities measured in Bielschowsky and Luxol fast blue images from human autopsy brain sections.

**Figure 7:**
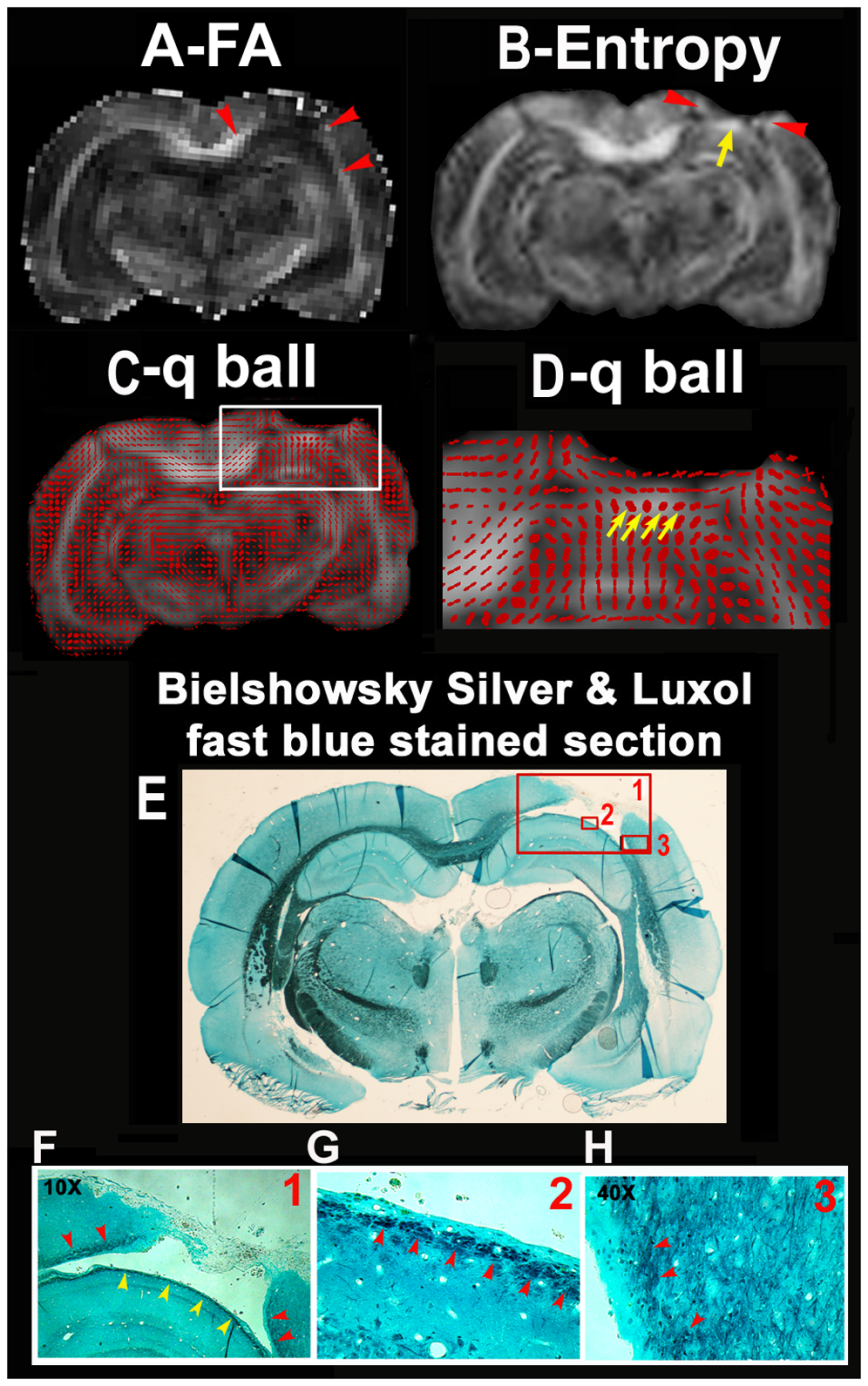
The FA (A), diffusion entropy (B), q-ball fiber orientation (C, D) maps overlayed onto entropy, and the Bielshowsky and Luxol fast blue immunoreactive staining images (E-H) measured from the fixed animal brain. The images in F, G are high magnification images from the box areas in image E as indicated by the numbers in the up right corner in the images of E and F.

Figure 7 shows *ex vivo* FA (A), entropy (B), and q-ball fiber orientation direction (FD) maps (C, D), and Bielshowsky and Luxol fast blue images (E-H) from the fixed animal brain 6 weeks after TBI. White matter reorganization after MSC treatment, confirmed by an increase in axons (C-H, black) and myelination E-H, blue), coincided with increases in FA (B, FA and D, red arrowheads) in the extended region of the corpus callosum surrounding the lesion. The entropy map revealed increased diffusion entropy not only at the boundary of the lesion as shown on the FA map but also at the base of the lesion (yellow arrow), where fiber crossings of axons were confirmed by the q-ball fiber orientation map (D, yellow arrows) and the Bielshowsky and Luxol fast blue images (F, yellow arrowheads and G, arrowheads).

## Discussion

In this study, diffusion entropy is investigated to detect axonal remodeling after injury. Our data demonstrated that diffusion entropy extracts more accurate structural details from brain tissues with crossing axonal bundles than does the conventional Gaussian model of DTI. The entropy method exhibits better dynamic range and distinction between different gray matter structures and CSF, without making any assumptions or modeling during the diffusion process. Our data also demonstrates that entropy strongly correlates with axonal density measured in human autopsy brain (r = 0.91), and is an important and sensitive method for detecting axonal remodeling during neurological recovery from animal model of TBI.

Brain injury, such as TBI, remains a leading cause of mortality and disability among children and young adults. Current research in brain injury has been restricted to acute neuroprotection treatment with a short treatment window [44], [45]. Effective interventions to enhance brain repair restorative cell-based and pharmacological therapies with extended therapeutic windows for experimental TBI have been developed[46], [47], [48]. In these investigations, functional recovery after TBI may be driven by neuronal and vascular remodeling[46], [47], [48]. Currently, the investigation of neuronal remodeling after brain injury has been dominated using traditional DTI such as FA and fiber tracking. As demonstrated in previous studies, FA has excellent potential for the assessment of white matter remodeling after TBI [6], [11], [49], [50], [51], [52]. However, when white matter fiber tracts cross, conventional DTI produces an anomalous result, showing inability to resolve more than one fiber direction and an overall lowering of FA despite the presence of highly-oriented tissue [18], [19], [29], [33], [35], [53]. The inability of conventional DTI to resolve multiple fiber directions and low FA derive from the assumption of Gaussian diffusion inherent to the tensor model, and different q-space DTI (qDTI)methodologies have been developed to address these issues [18], [19], [29], [33], [35], [53]. Comparing the successful in developing Q-DTI to resolve multiple fiber directions [18], [19], [29], [34], there are few published studies to target the issue related to low FA in areas with multiple fiber directions [33], [35], [53], [54] and no investigation has been published in white matter remodeling after brain injury, such as TBI, using diffusion entropy MRI. Our data demonstrated that diffusion entropy is more sensitive to axonal density than orientation and can detect axonal remodeling with random fiber orientation after TBI. We determined that entropy is superior to FA in detecting white matter reorganization with prominent crossing axons; moreover, it is sensitive to early stages of white matter reorganization (more crossing fibers), and increases significantly compared with the relatively low FA. However, both entropy and FA show a similar pattern if the white matter bundle is well organized in a single direction. A combination of entropy and FA may provide information about the stage of white matter reorganization in the injured brain, e.g., increased entropy in the absence of elevated FA would represent an early stage of recovery as typified by random crossing fibers, while increased FA would identify more mature linear fibers.

Entropy measurement can overcome the limitations of the conventional Gaussian model of DTI in detecting structural changes in gray matter and white matter containing crossing axons. Although conventional DTI based on the Gaussian model has become an important clinical measurement in detecting WM changes, it can not detect structural changes in gray matter due to its associated very small dynamic range, and can also produce large errors in detecting WM with crossing axons [19], [29], [34]. When white matter fiber tracts cross, conventional DTI shows an overall lowering of FA despite the presence of highly-oriented tissue. The inability of conventional DTI to resolve multiple fiber directions derives from the assumption of Gaussian diffusion inherent to the tensor model [18], [24], [55]. The conventional tensor model assumes Gaussian diffusion, and a Gaussian function has only a single directional maximum. Consequently, the conventional tensor model cannot capture multidirectional diffusion. The MR diffusion signal has significant multimodal structure, in clear disagreement with the conventional tensor model [19], [29], [34], [55]. However, current Q-space DTI research has mainly focused on fiber orientation detection and less on the quantitative determination of the density in complex brain tissues. In the current study, quantitative measurement of entropy shows great potential in characterizing complex brain tissues. In contrast to FA (4–7%), entropy exhibits much larger differences (from 20–24%) between CSF and different gray matter structures, e.g., putamen, caudate nucleus and cortex. The relative values of entropy in gray matter structures with respect to the corpus callosum were approximately triple compared with FA. This enhanced contrast provided by the entropy map provides detailed visible structural information and is able to detect structure changes in gray matter. Most importantly, our data demonstrated that entropy strongly correlated to axonal density and was less dependent upon orientation. Therefore, entropy could provide more accurate information about structural changes than conventional DTI in gray matter and WM with crossing axons (during disease processes). Our data demonstrated that entropy is highly correlated with axonal density. High entropy value in CC was induced from the high density of axons, confirmed with histology data, and from the unidirectionality of axons which causes a more even distribution of attenuation values in each direction of gradients, as demonstrated in Fig 4. Comparing with CC, reduced entropy in other regions could arise predominately from lower axonal density and also from multiaxonal orientation. Therefore, the non model based entropy approach can reduce the modeling induced errors to derive a measurement more close to axonal density compared to FA, since the lower FA value in FW is attributed to its inherent modeling error especially in the fiber crossing regions.

The number of diffusion directions affects entropy values. Although entropy values could be more stable and accurate with increased number of directions, it is more time consuming and less tolerable for patients. The optimum number of directions is determined by obtaining stable and reliable entropy values close to that measured with a large number of directions, such as 90, with minimum scan time or minimum number of directions. The entropy in CSF decreases with the increase in number of directions. CSF should have an entropy value close to 0 due to evident lack of structure. The measured entropy values in CSF are most likely due to image noise. The increased number of directions will increase the signal and decrease noise to derive decreased entropy. The CC has more dense unidirectional brain structure and therefore higher entropy values. The entropy value should be increased and then be stabilized after number of directions could provide proper information content and signal to noise ratio. Our data indicate that 55 directions may be an optimum number for diffusion directions to have relatively stable entropy in white matter and CSF. Also, there is no significant difference in entropy values between 55 and 90 directions in all the ROIs.

Distribution of diffusion attenuation reflects amplitude of entropy. Mobility of water molecules along different gradient directions in brain can be evaluated using the attenuation of the MRI signal [13], [23]. Depending on the direction of the applied gradient, attenuation values change due to anisotropic tissue structures. As demonstrated in Figure 4, a lack of brain structure, such as CSF, has isotropic diffusion, yielding higher probability of occurrence along all diffusion directions, and has higher occurrence probabilities, which therefore results in lower entropy values close to zero. In contrast to CSF, CC has higher axonal density, a unidirectional axonal bundle, and therefore exhibits a more even distribution of diffusion attenuation (Figure 4) resulting a higher entropy. Simply based on attenuation values brain, structures in brain can be differentiated [36]. Although entropy exhibits a high agreement with axonal density, it is also affected by the geometry of tissue structure.

Entropy is superior to FA in detecting white matter reorganization with prominent crossing axons. The entropy is sensitive to the early stage of white matter reorganization with more crossing fibers, where the entropy significantly increases compared with the relatively low FA. However, the entropy shows a similar pattern as FA if the white matter is well organized to a single direction. The combination of entropy and FA may provide information about the stage of white matter reorganization in the injured brain, with increased entropy alone (without FA elevation) representing an early recovery stage of white matter reorganization with crossing fiber, while the increased FA identifies more mature linear fibers.

## Conclusion

This study investigated diffusion entropy in evaluating different brain tissues and its ability in detecting axonal remodeling after injury. Diffusion entropy resolves crossing fibers induced error without making any model assumption on the diffusion process, and detects axonal remodeling with crossing axonal bundles. Our results show high agreement (r = 0.91) between entropy and axonal density and superior image contrast in entropy compared with that in FA. Compared with FA, entropy may provide improved quantitative biomarkers of diseases that affect brain structures involving gray matter and white matter with crossing axons.
